# Effect of beak trimming and feather spraying with bitter taste compound on feather pecking and welfare of Muscovy ducks

**DOI:** 10.1038/s41598-025-01371-x

**Published:** 2025-05-21

**Authors:** Eman Hefnawy, Ahmed Sabek, Saeed El-laithy, Souad Ahmed

**Affiliations:** Department of Hygiene and Veterinary Management, Faculty of Veterinary medicine, Benha University, Moshtohour, 13736, Benha city, 13518 Egypt

**Keywords:** Muscovy ducks, Beak trimming, Almond oil, Bill morphology, Welfare, Feather pecking, Biochemistry, Physiology, Zoology

## Abstract

The current study was conducted to compare the effect of beak trimming and feather spraying with bitter taste compound (almond oil) on feather pecking behavior and duck welfare. A total of 63 healthy male 2-weeks-old Muscovy ducklings were randomly allocated to 3 groups. The first group was the control group (no beak trimming and no feather spraying), ducks in the second group were trimmed by scissors at 3 weeks of age, and birds in the third group were sprayed with bitter almond oil weekly from the age of 3 weeks. Growth performance parameters were evaluated. Some behavioral patterns were recorded by using focal sampling. Feather condition score and serum cortisol level were evaluated. At the end of the experiment, bills were collected for histopathological examination. The results showed that beak trimming with scissors had no negative effects on Muscovy ducks’ growth performance and significantly lowered feather pecking bouts resulting in good feather conditions. Feather spraying with bitter almond oil had adverse effects on growth performance, obviously increased feather pecking resulted in deterioration of feather quality and markedly increased the level of cortisol *(p < 0.001)*. Bill morphological analysis with hematoxylin and eosin (H&E) and S100 stains illustrated that the trimmed beak had an increase in the amount of connective tissue (scar tissue formation), numerous blood vessels, fewer nerve bundles, and no neuroma formation. In the Muscovy ducks’ sector, beak trimming with scissors at 3 weeks of age is good practice to control feather pecking and cannibalism without adverse effect on the welfare of ducks.

## Introduction

Feather pecking (FP) is a non-aggressive pecking activity in which birds peck at or pull off the feather of another bird. Depending on the severity and negative effects on birds’ feather pecking can be classified as mild and severe^1^. Mild FP is pecking at the plumage of another bird without excessive force and pulling out of feathers; it is considered a natural habit that contributes to social exploration so, it does little or no damage to the other bird^2^. Feather pecking impairs the welfare, performance, productivity, and health of birds by reducing feed intake and nutrient utilization. Forceful falling and pulling of feathers as well as ingestion of pulled feathers are symptoms of severe feather pecking. It is harmful in nature causing, feather damage, feather loss, sores, pain, cannibalistic pecking, and death in certain cases^3^. The precise origins of, and solutions for feather pecking remain inadequately comprehended. Numerous factors have been recognized as contributors to the emergence of feather pecking and cannibalism within poultry flocks including genetics, number of birds per group, light intensity, diet^4^, and light color^5^.

Recently feather pecking can be controlled by different ways as bill trimming that has been applied to reduce the damage resulted from feather pecking that mostly leads to cannibalism^6^. Beak trimming can be carried out by different methods such as using sharp scissors or hot blades. Some chemical compounds with a bitter taste sprayed on bird plumage contributes to feather pecking prohibition through taste aversion learning^7^. Another way to prevent feather pecking is environmental enrichment through providing birds with aids that improved its behavior and welfare as novel object and dust bathing^8^.

Beak trimming is the removal of one-quarter to one-third of the upper beak or both upper and lower beaks of birds^9^ or the removal of sharp upper and lower tips of the beak to reduce peck injuries and deaths within a group of birds^10^. Beak trimming is acutely painful and occurs with several different methods that can be classified into four major groups: mechanical, hot blade, electrical, and infrared. The practice of beak trimming has been debated in animal welfare circles as it causes pain and stress to the birds^11,12^. Applying a bitter compound to the wings reduced aggressive pecking among birds, and the effect of the bitter taste lasted for 1–2 weeks, so it is possible to prevent feather pecking problems by utilizing the bitter taste sensation. Spraying downy feathers in young chicks can provide a taste aversion for feathers in adult chickens and help in protection against severe FP^7^.Odor can act as discriminative stimuli for taste-avoidance learning in birds, and it is more powerful and helps in controlling pecking behavior in birds.

A previous study revealed that chicks learned to avoid almond-smelling water and drank water that did not smell of almond. A memory test also showed that chicks had a higher avoidance of almond odor after 24 h as their memory of almond was durable^13^. Almond oil increased the rate at which chicks learn to avoid unpalatable solutions, and can produce a taste aversion through its odor^14^. The effect of beak trimming on feather pecking was widely described in much previous studies in different poultry species as Muscovy ducks^15^, hens^16^,and quails^17^. Additionally, spraying the feather with bitter taste material was investigated in hens^7^. Currently, there is no evidence assessing the impact of bitter taste compounds on feather pecking and cannibalism control in Muscovy ducks. This study was undertaken to examine the effects of bill trimming vs. the application of a bitter-tasting substance (almond oil) on the performance, welfare, feather pecking behavior, and beak morphology of Muscovy ducks.

## Materials and methods

### Birds and management

A total of sixty-three healthy male Muscovy ducklings aged 2 weeks with an average body weight of 286.66 ± 8.53 g were purchased from a private local company in Egypt. Birds were housed in three symmetrical pens; each pen’s dimensions were 3.75 m, 3.60 m, and 3 m for length, width, and height respectively. The pens had been previously cleaned and disinfected. During the experimental phase, the average room temperature was 28.55 ± 0.20 °C, the relative humidity was 65.19 ± 0.45%, and the photoperiod was 16 h. light and 8 h dark. All ducklings were vaccinated against avian influenza and fowl cholera at the age of 4 and 6 weeks, respectively. Feeders and drinkers were equally distributed in the pens, and clean, fresh water was available throughout the day. From 2 to 4 weeks old, a starter diet contained 22% crude protein was given^18^, followed by a grower diet contained 19% crude protein as recommended by^19^ from 5 to 10 weeks old.

## Experimental design

A total of 63 healthy male Muscovy ducklings aged 2 weeks were randomly assigned to 3 groups; each group contained 21 birds that were divided into 3 replicates with 7 birds each. Each group was housed in a separate pen that was divided into three parts using wooden barriers, one part per replicate. Each part’s dimensions were 170 cm in length and 120 cm in width. The first group was kept as a control in which ducks were not subjected to the beak trimming or feather spraying; the birds in the second group were exposed to beak trimming with scissors at 21 days old by removing the upper hock of beak according to^4^. The birds in the third group were subjected to feather spraying at 3 weeks old with a bitter almond oil solution. The feathers were sprayed with 1% bitter almond oil solution that is prepared by dilution of oil with ethanol first (1:2), then mixing with water to make a solution according to^20^.The study was conducted during the growing period of Muscovy ducks from 2 to 10 weeks old from the beginning of May to the end of June 2024.

## Growth performance parameters

The initial and the final body weights of all the birds in each group were measured. Weekly feed intake was determined by deducting the leftover quantity from the weekly amount fed to each group of birds. The amount of feed consumed divided by the total number of birds is the feed intake per bird, expressed in grams. Body weight gain (BWG) is the difference between the final body weight and the initial body weight. Feed intake divided by body weight gain is the feed conversion rate (FCR).

## Behavioral observation

The behavioral observations were started when the ducks were 4 weeks old after giving them 1 week for adaptation to the place and 1 week for adaptation to beak trimming and feather spraying. The behavioral patterns of 15 birds per group (5/ replicate) were recorded 3 days a week, twice per day, at 9.00–10.00 am and 2.00–3.00 pm. Each bird´s behavioral patterns were observed by focal observation for a continuous 3 min, with a total observation time of 15 min per replicate per group in the morning and in the afternoon. All observations were conducted by one observer who presented at all measurement points of the experiment. The observed behaviors were mentioned in detail in our previous study^5^.

## Feather condition score

To evaluate the effect of beak trimming and feather spraying with bitter almond oil, feather condition score was performed. The feather condition of all birds per group was determined weekly using a score scale as follows: score 0: good, indicates full feathering; score 1: moderate, indicates slight feather pecking, slight damaged areas less than 1cm^2^; score 2: bad, indicates severe feather pecking, bleeding, sever damaged areas more than 2 cm^2^ ^21^.

### Blood sampling and hormonal analysis

At the end of the study, 5 ducks from each treatment were selected randomly and were sacrificed for blood sampling. Slaughtering was done using a sharp knife that made a single cut across the neck, cutting the carotid arteries, jugular veins, esophagus, trachea, and the connective tissues of the neck without pre-slaughter anesthesia. Knife sharpness is very important as it promotes better bleeding and reduces discomfort and anxiety in birds by inducing rapid unconsciousness.

3 ml of blood was collected in a clean, sterilized, labeled tube without anticoagulant, and centrifuged at 3000 rpm for 15 min. The serum was separated and kept at -20 °C until analysis. In the current study, cortisol was measured by ELISA using (Cortisol II, cobas^®^) kits according to the manufacturer’s recommendations.

## Bill morphological analysis

Five ducks per treatment were randomly selected at the end of the experiment and were slaughtered for morphological bill analysis. Bills were cut with scissors and beaks were collected (its length from tip till nares) in plastic jars. The beaks were preserved in a 20% buffered formalin solution then decalcified in 10% formic acid for one week. Post-decalcification preserved ducks’ beak tissues were processed in an automated tissue processor. The processing consisted of an initial 2 steps fixation and dehydration. Fixation comprising tissue immersion in 10% buffered formalin for 48 h, followed by removal of fixative in distilled water for 30 min. Dehydration was then carried out by running the tissues through a graded series of alcohol (70%, 90%, and 100%). The tissue was initially exposed to 70% alcohol for 120 min followed by 90% alcohol for 90 min and then two cycles of absolute alcohol, each for one hour. Dehydration was then followed by clearing the samples in several changes of Xylene. It consisted of tissue immersion for an hour in a mixture comprising 50% alcohol and 50% Xylen, followed by pure Xylene for one and a half hour. The samples were then impregnated with molten paraffin wax, then embedded and blocked out. Paraffin Sects. (4–5 μm) were stained with H&E^22^ stained sections were examined for the presence of nerve bundles and nerve ganglion distribution beside accompanying tissue changes including degeneration, necrosis, apoptosis, inflammation, proliferation and any other pathological changes and the tissue sections were examined by immuno-histochemical methods by using tissue marker (S100) that is highly specific and sensitive to react with nerve bundles and nerve ganglion distribution. Photos were taken at magnification (H&E and S100 × 100, 200**)**.

### Statistical analysis

SPSS version 22 was used to analyze the data. Data were analyzed using analysis of variance (ANOVA) of CRD treatments. Means and standard error means were used to present the data. *P* ≤ 0.05 were used to declare the data to be different.

## Results

### Growth performance

The results revealed that the effect of beak trimming and feather spraying with bitter almond oil had no significant effect on the initial body weight, final body weight, body weight gain, and FCR of ducks. The feather spraying group displayed the lowest feed intake when compared to other treatments *(P = 0.05)* (Table 1).

**Table 1:** Effect of beak trimming and feather spraying with bitter almond oil on the growth performance parameters of Muscovy ducks during growing period.

Growth performance parameters	Groups			
	Control	Beak trimming	Feather spraying	*P* – Value
Initial body weight (g)	288.33 ± 7.08	287.00 ± 7.08	290.33 ± 7.08	0.945
Final body weight (g)	3428 ± 181.91	3090 ± 181.91	2885 ± 181.91	0.116
Body weight gain (g)	3140 ± 207.71	2803 ± 207.71	2595 ± 207.71	0.251
Feed intake (g)	7407^a^ ± 312.40	6699^ab^ ± 312.40	6204^b^ ± 312.40	0.058
Feed conversion rate (FCR)	2.36 ± 0.08	2.39 ± 0.08	2.41 ± 0.08	0.922

Least square means (± SE) with different superscripts letters in the same row are significantly different at *p* ≤ 0.05.

### Behavioral patterns

Behavioral patterns as affected by beak trimming and feather spraying with bitter almond oil are shown in Table 2. Drinking and object pecking were significantly affected as the highest frequency of drinking and object pecking were recorded in control group followed by beak trimming group while the lowest drinking frequencies were observed in ducks sprayed with bitter almond oil (*P = 0.01* and *0.02*) for drinking and object pecking respectively.

**Table 2:** Effect of beak trimming and feather spraying with bitter almond oil on some of behavioral patterns of Muscovy ducks during growing period.

Behavioral patterns Frequency	Groups			
	Control	Beak trimming	Feather spraying	*P* - Value
Feeding	3.03 ± 0.35	2.60 ± 0.35	2.00 ± 0.35	0.117
Drinking	5.90^a^ ± 0.51	4.73^ab^ ± 0.51	3.63^b^ ± 0.51	0.011
Sitting	14.90 ± 0.70	13.33 ± 0.70	13.70 ± 0.70	0.267
Walking	6.56 ± 0.53	6.40 ± 0.53	5.20 ± 0.53	0.144
Standing	7.66 ± 0.68	8.16 ± 0.68	6.90 ± 0.68	0.425
Preening	10.46 ± 0.91	8.76 ± 0.91	8.70 ± 0.91	0.309
Wing & Leg stretch	2.96^a^ ± 0.36	1.76^b^ ± 0.36	2.30^ab^ ± 0.36	0.059
Head shaking	2.30^a^ ± 0.31	1.40^b^ ± 0.31	1.20^b^ ± 0.31	0.035
Tail wagging	4.73 ± 0.46	4.23 ± 0.46	3.93 ± 0.46	0.477
Feather pecking	1.23^b^ ± 0.45	0.83^b^ ± 0.45	3.36^a^ ± 0.45	< 0.001
Litter scratching	5.36^a^ ± 0.55	3.13^b^ ± 0.55	3.96^ab^ ± 0.55	0.018
Object pecking	3.96^a^ ± 0.33	1.33^ab^ ± 0.33	0.86^b^ ± 0.33	0.028

Least square means (± SE) with different superscripts letters in the same row are significantly different at *p* ≤ 0.05.

Feeding, sitting, standing, walking, preening, and tail wagging frequencies were not significantly affected by beak trimming and feather spraying. Ducks in control group had higher frequencies of wing, leg stretch and head shaking than beak trimming and feather spraying groups *(P = 0.05 and P = 0.03)* for wing, leg stretch and head shaking, respectively). Litter scratching was observed more in control group than feather spraying and beak trimming groups *(P = 0.01)*. Ducks in bitter almond oil spraying group showed more frequency of feather pecking than ducks at beak trimming and control groups *(P* < 0.001).

### Feather condition score

As indicated in Table 3, ducks in all groups showed a good feather condition score (score 0) from the 3rd to the 5th week of age. At 6 to 7 weeks old, ducks that sprayed with bitter almond oil displayed a moderate feather condition score (score 1). At the age of 8, 9, and 10 weeks control and sprayed ducks showed a moderate feather condition score (score 1) compared to beak trimmed ducks that showed a good feather condition score (score 0).

**Table 3:** Effect of beak trimming and feather spraying with bitter almond oil on feather condition score of Muscovy ducks during growing period.

Feather score/week	Groups		
	Control	Beak trimming	Feather spraying
Week 3	0	0	0
Week 4	0	0	0
Week 5	0	0	0
Week 6	0^b^	0^b^	1^a^
Week 7	0^b^	0^b^	1^a^
Week 8	1^a^	0^b^	1^a^
Week 9	1^a^	0^b^	1^a^
Week 10	1^a^	0^b^	1^a^

Scores with different superscripts letters in the same row are significantly different at *p* ≤ 0.05.

### Serum cortisol level

The current results revealed a significant difference in cortisol level among different treatment groups. Ducks sprayed with bitter almond oil had the highest cortisol concentration followed by beak trimming and control treatments respectively (P < 0.001) (Table 4).

**Table 4:** Effect of beak trimming and feather spraying with bitter almond oil on Serum cortisol level of Muscovy ducks during growing period.

Item	Groups			
	Control	Beak trimming	Feather spraying	*P* – Value
Serum cortisol(ug/dl)	1.17^c^ ± 0.05	1.71^b^ ± 0.05	2.31^a^ ± 0.05	< 0.001

Least square means (± SE) with different superscripts letters in the same row are significantly different at *p* ≤ 0.05.

### Bill morphological analysis

**Fig. 1:**
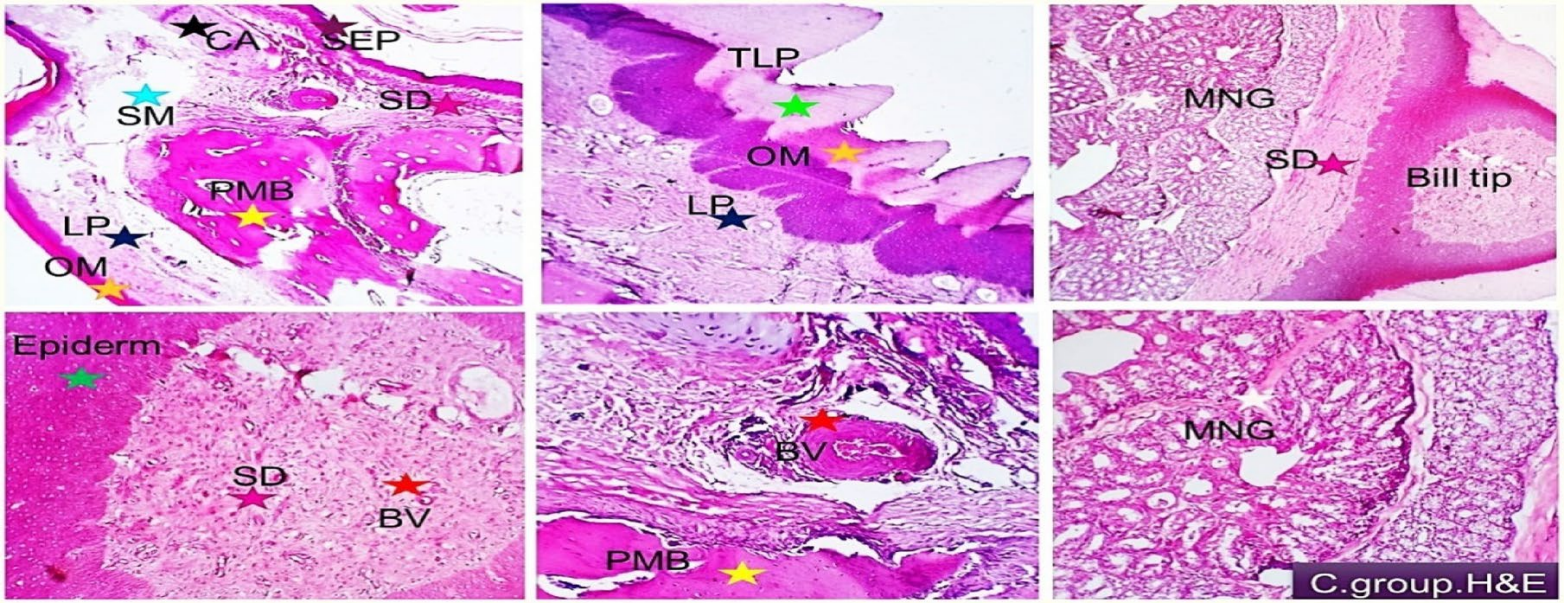
Histological and immune-histochemical findings of the beak of control group. Demonstrating two surfaces (cutaneous and oral mucosal surfaces). The former constitute of highly keratinized skin epidermal tissue (SEP, brown asterisk) followed by dermal fibro-elastic layer (SD, purple asterisk) comprising many types of sensory mechano-receptors, beside cavernous blood spaces (BVred asterisk) followed by premaxillary bone tissue (PMB, yellow asterisk) covered by cartilaginous cap (CA, black asterisk). The oral side showed tick keratinized mucosa (OM, yellow asterisk) with sharp tooth like serrated projections (TLP, green asterisk) This layer followed by the lamina propria (fibrous connective tissue layer containing sensory mechano-receptors and vascular structures, LP, dark blue asterisk). Premaxillary cartilaginous bone structure (PMB) is seen between the lamina propria and the submucosa which enclose mucinous maxillary nasal glands (MNG, white asterisk), loose connective tissue and vascular structures. H&E, S100 × 100, 200.

**Fig. 2:**
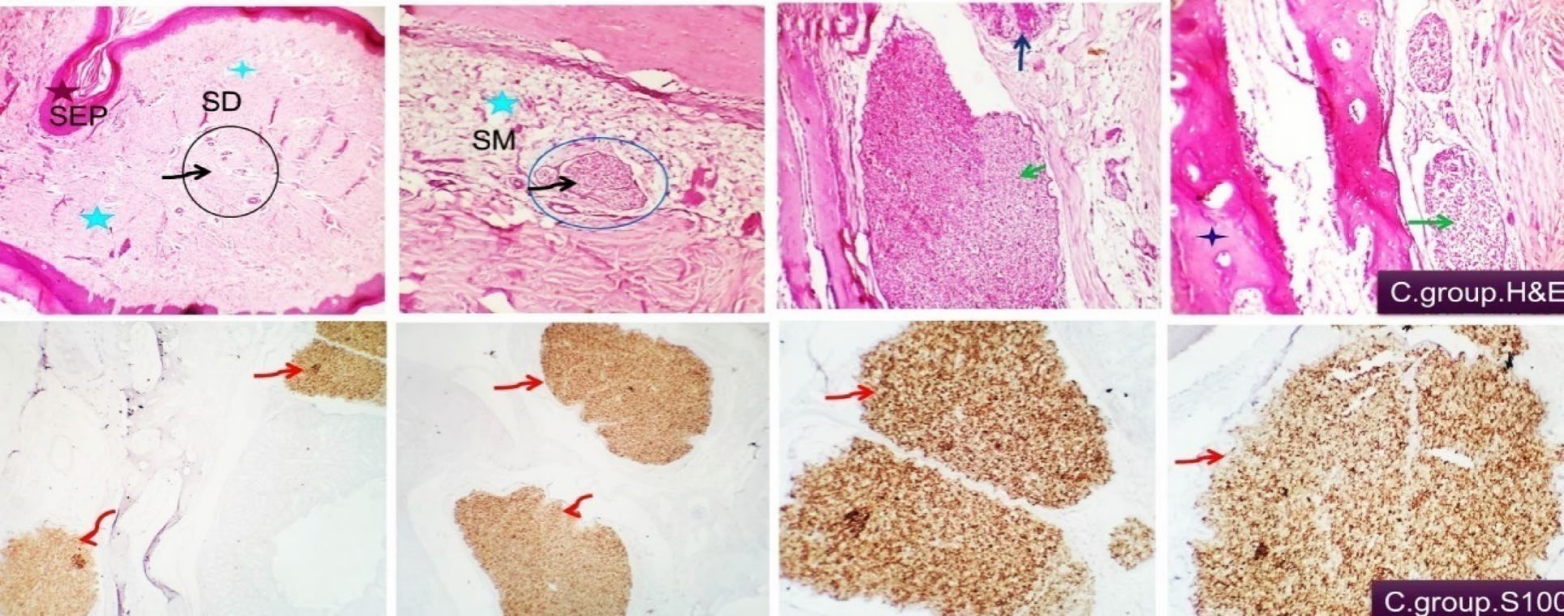
Histological and immune-histochemical findings of the beak of control group showing nerve bundles. Demonstrating the structural distribution of different sizable nerve bundles at the dermal tissue (black arrow and circle), oral submucosa (black arrow and blue circle), peri-glandular (green and dark blue arrows) and pre-maxillary (green arrow, dark blue asterisk). S100 immuno-positive nerve bundles are marked by red arrows and are seen in pre-maxillary, in the skin dermal tissue and the oral submucosa. H&E, S100 × 100, 200.

Examined tissue sections from duck’s beaks in all treatments revealed two surfaces (cutaneous and oral mucosal surfaces). In the control group, the former constitutes highly keratinized skin epidermal tissue followed by dermal fibro-elastic layer comprising many types of sensory mechano-receptors (Ruffini corpuscles, Merkel corpuscle, Grandry corpuscle, and Herbst corpuscle) beside cavernous blood spaces followed by premaxillary bone tissue covered by cartilaginous cap. The oral side showed thick keratinized mucosa with sharp tooth like serrated projections. This layer is followed by the lamina propria (fibrous connective tissue layer containing sensory mechano-receptors and vascular structures). Premaxillary cartilaginous bone structure is seen between the lamina propria and the submucosa which enclose mucinous maxillary nasal glands, loose connective tissue, and vascular structures (Fig. 1). The connective tissue and the intervening blood vessels and capillaries were of average distribution, amounts, normal histo-morphology and free from degenerative, apoptotic or necrotic changes. Moderate distribution of nerve bundles of variable sizes at different locations. Specific sensory nerve cells as Herbst corpuscles were also demonstrated (Fig. 2 upper). Immuno-histochemical examination showed that the large and small nerve bundles and the neuronal ganglion cells were highly reactive to S100 and the distribution in different parts of the beak tissue was highly visualized. Nerve cells were seen in pre-maxillary bone, in the dermal tissue, and in the oral sub mucosa (Fig. 2 lower).

**Fig. 3:**
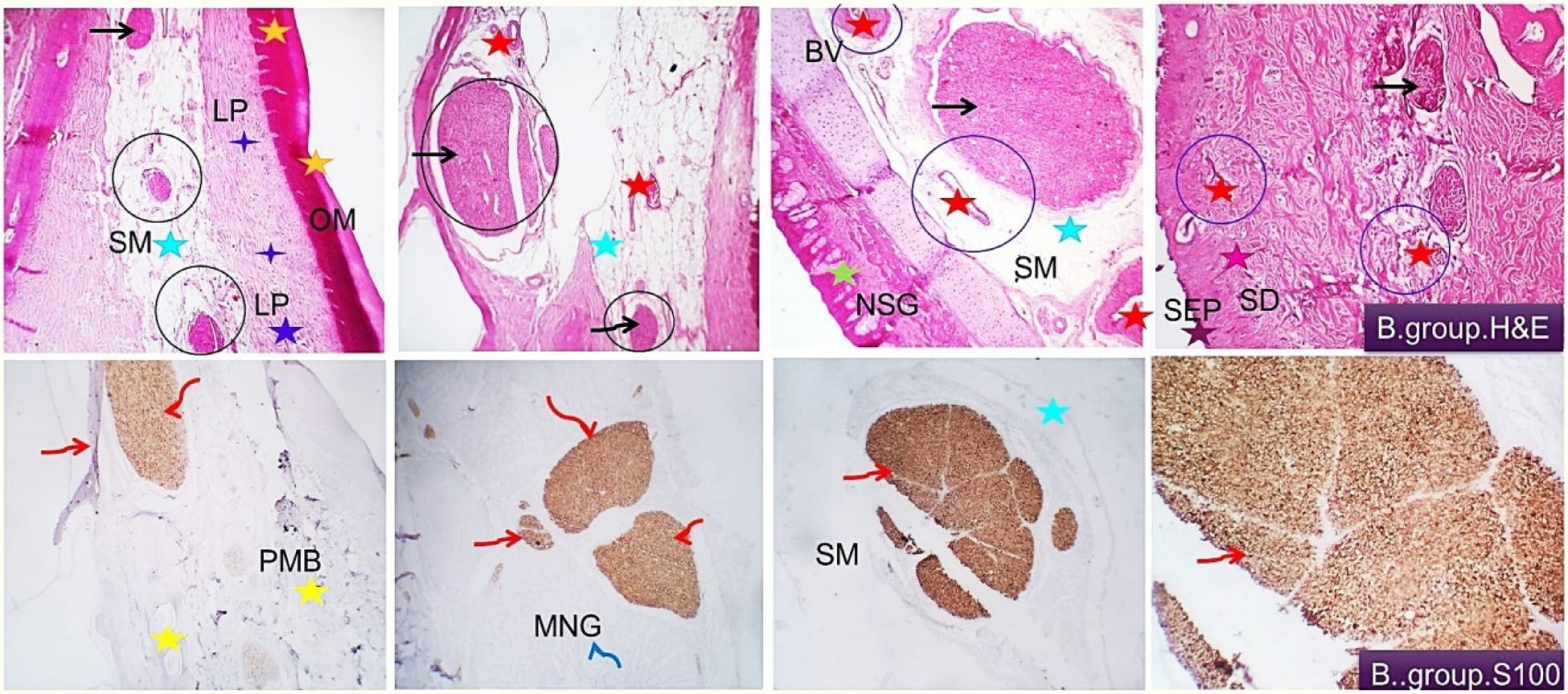
Histological and immune-histochemical findings of the beak of beak trimming group. Demonstrating moderate distribution of nerve bundles of variable sizes at different locations including the superficial sup-epithelial tissue (mucosal lamina propria (LP, dark blue asterisks) and cutaneous dermal tissue (SD, purple asterisks), among the loose fibrous interstitial tissue of the deep dermis or sub mucosa (SM, light blue asterisks, black circles and arrows) and around the maxillary nasal glandular tissue (NSG, green asterisks, black arrow). Disappearance of the epidermal tissue (SEP, brown asterisk) and hyperplasia of the dermal fibro-elastic layer which comprises many blood vessels and cavernous blood spaces (blue circles, red asterisks) are seen. Immuno-histochemical investigation showing a highly reactive nerve bundle to S100. Their distribution at different parts of the beak tissue is highly visualized (around and in the premaxillary (PMB, yellow asterisk, red arrows), around the mucous nasal glands (MNG, blue and red arrows) and in the submucosa (SM, light blue asterisk, red arrow). A few nerve bundles appear weakly reactive to the used marker (black asterisk).H&E, S100 × 100, 200.

In the beak trimming ducks, the former showed disappearance of the epidermal tissue, and the dermal fibro-elastic layer was hyperplastic and comprising many types of sensory mechano-receptors beside many blood vessels and cavernous blood spaces followed by premaxillary bone tissue covered by cartilaginous cap. Nasal sinus epithelial and subepithelial mucous glands were seen. The oral side showed the same findings that were previously observed in control group beaks (Fig. 3 upper). The connective tissue of the submucosa was edematous and comprising many blood vessels. Moderate distribution of nerve bundles of variable sizes at different locations. Specific sensory nerve cells such as Herbst corpuscles were also demonstrated. Immuno-histochemical investigation pointed out the large and small nerve bundles and the neuronal ganglion cells were highly reactive to S100 and their distribution in different parts of the beak tissue was highly visualized. A few nerve bundles were weakly reactive to the used marker. They may constitute newly formed and regenerated nerve fibers. Nerve fibers were seen, in the dermal tissue, in the oral sub mucosa, around the naso-sinus glands and pre-maxillary. (Fig. 3 lower)

**Fig. 4:**
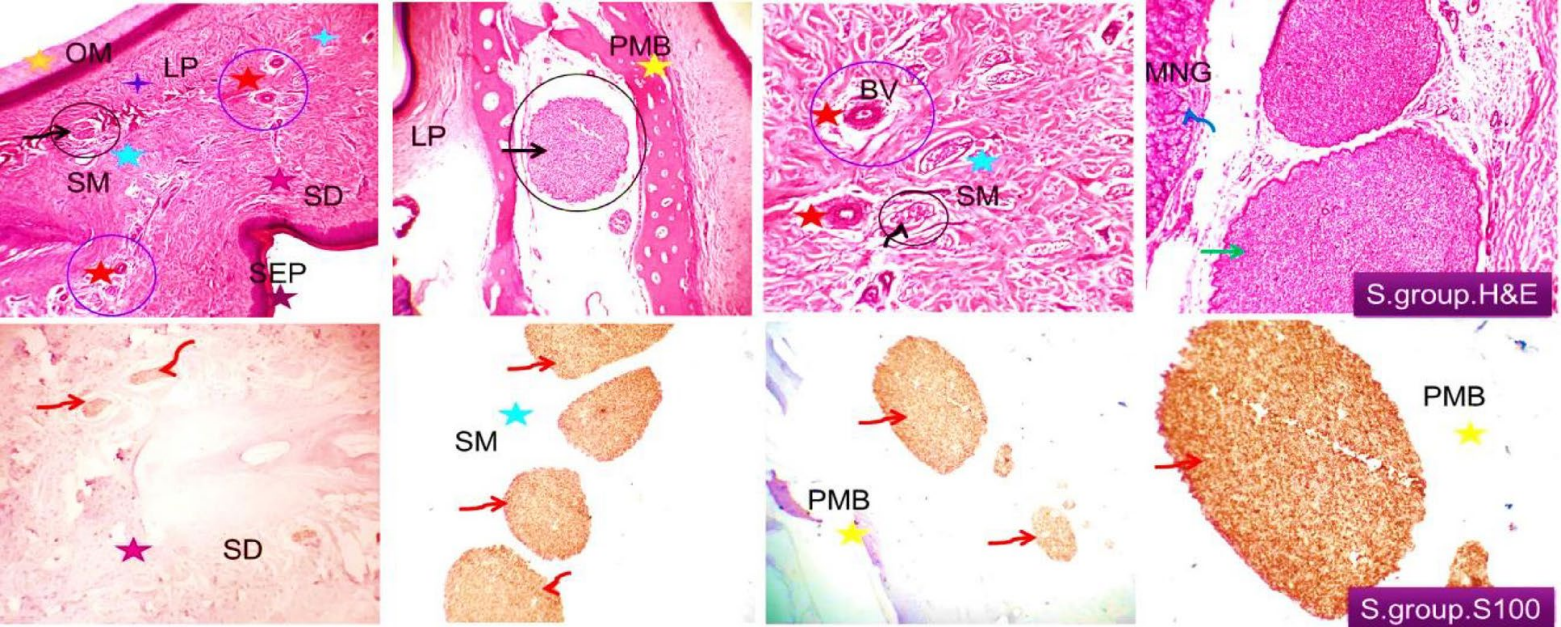
Histological and immune-histochemical findings of the beak of bitter almond oil spraying group. Demonstrating an average distribution of different sizable nerve bundles at the dermal tissue, inter-osseous, in the oral submucosa (black arrow and circle) and peri-glandular (green and dark blue arrows) and pre-maxillary (green and dark blue arrows). Prominent connective tissue bundles are seen (black asterisks). The vascular structures appear prominent (blue circles and red asterisks). S100 immuno-positive nerve bundles are marked by red arrows. They are seen in the skin dermal tissue (SD, purple asterisk), oral submucosa (SM, light blue asterisk) and around the pre-maxillary bone (PMB).H&E, S100 × 100, 200.

The tissue sections from duck’s beaks of bitter almond oil sprayed group revealed the same characteristics of the control group ducks (Fig. 4 upper). The connective tissue was of average distribution and of normal histo-morphology and free from degenerative, apoptotic or necrotic changes as same as control ducks while it was more prominent. The vascular structures were more prominent compared with the control group although their distribution was comparable. Moderate distribution of nerve bundles of variable sizes at different locations. Specific sensory nerve cells such as Herbst corpuscles were also demonstrated. Immuno-histochemical investigation pointed out the same findings that were observed in control ducks. There were no significant countable or structural changes in the nerve fibers of this group as compared with the control one (Fig. 4 lower).

## Discussion

The final body weight was not significantly different among the 3 groups. However, the control ducks showed higher body weight than trimmed and bitter almond sprayed ducks. This may be attributed to the effect of beak trimming and feather spraying that act as stress factors. Ducks before swallowing and tasting food using taste buds on the tip of the beak and used their bill stump not only for food transport but also for food discrimination. So, removing the bill stump could interfere with food identification and the feeding behavior^4^. Our findings are in agreement with^15^ who reported that trimmed Muscovy ducks with scissors had a lower body weight than control ducks. Also our results agree with^23^ who found that beak trimmed birds had a lower body weight when compared to control group. Previous studies reported that bill trimming lowered the body weight of birds^24,25^. In contrast scissor-treated mule ducks showed higher final body weight than control ducks^26^. In large white turkey, the average body weight was not affected by beak trimming^27^. The body weight of ducks remained unaffected by beak reduction or bitter almond oil application, and consequently, body weight gain was also uninfluenced, corroborating findings from prior research^4^.

Conversely beak trimming with scissors had a significant increase on body weight gain of mule ducks (*P* < 0.05) than control^26^. In the current study control ducks consumed more feed than trimmed and bitter almond oil sprayed ducks. Feed consumption of birds with intact beaks was statistically higher than trimmed birds^28,29^. Beak trimming reduced feed intake and subsequent growth rate of Single Comb White Leghorn hens^16^^30^ found that trimmed birds obviously consumed less feed than the control birds.

Changes in feed intake after beak trimming, regardless of the trimming method or bird age were associated with an increase in the presence of pain and discomfort produced by tissue, neuron, or sensory receptor injury^31^. This leads to a lack of motivation to search for food^28^. There was no significant treatment difference in total feed conversion ratio during the experiment. In contrast to the current findings. Bitter almond oil sprayed ducks had low body weight, body weight gain, and feed intake. This may be attributed to the bitter taste of almond oil that the ducks obtained from feather in their beaks resulted in an irritation and taste aversion from feed so, it increased stress upon the birds leading to an increase in feather pecking and feather eating.

Beak trimming with scissors and spraying with bitter almond oil showed obvious differences in behavioral bouts that affect the welfare of Muscovy ducks. Feeding frequency did not differ significantly between different treatments. Our results are in agreement with^15^ who found no significant difference in feeding frequency between control and bill trimmed Muscovy ducks. There was no long-term effect of bill trimming on the behavior of laying hens^25^.

In contrast, feeding frequency was significantly increased in trimmed mule ducks with scissors than control mule ducks^26^ unlike^29,32,33^ who reported a significant reduction in feeding bouts occurred in trimmed birds as compared to control ones.

The practice of beak trimming in poultry causes discomfort and tension. It has an impact on feed consumption in the days after the beak trimming resulting from the wound that is made by the process^24^. Beak trimming resulted in reduction in bird behaviors like feed intake^12^. Reduction in feeding of bitter almond oil group may be attributed to the taste aversion of the bitter oil that affects feed intake of ducks.

Drinking bouts differed significantly between control, beak trimmed and bitter almond oil sprayed ducks. It was increased in control ducks than trimmed and sprayed ducks. Our data agrees with^26^ who found drinking frequency increased significantly in control mule ducks than trimmed mule ducks. In contrast, beak trimming showed no significant effect on drinking frequences of Muscovy ducks^15^. Reduction in drinking behavior in beak trimmed and sprayed ducks may be attributed to decrease in the bill related behaviors in beak trimmed birds and the stress resulted from the taste of the almond oil.

Our findings revealed that no significant treatment changes in sitting frequency of Muscovy ducks. These results agree with the findings of^26^ who observed that sitting bouts of trimmed mule ducks with scissors did not differ significantly from control mule ducks. On the other hand, resting frequency increased immediately after trimming of Muscovy ducks with scissors^4^. Also, there are no notable changes observed in walking and standing frequencies between control, beak trimmed, and almond oil sprayed ducks. Our data disagrees with^26^ who reported that standing and walking bouts were obviously different between control and trimmed mule ducks. No statistical change in preening and tail wagging bouts was recorded between the different treatments ^26^ found that beak trimming produces a significant change in preening bouts between trimmed and control mule ducks also, Beak trimming decreased preening just after trimming then gradually increased till reach to non-significant difference between trimmed and control ducks at the end of the experiment^4^. In the current study sitting, walking, standing, preening, and tail wagging were not significantly affected by beak trimming and bitter almond oil spraying other factors may be responsible for change in these activities such as litter type and or light color, intensity, and duration. Head shaking and wing, leg stretch are comfort behaviors in the current study these behaviors obviously higher in control than beak trimming and almond oil sprayed ducks. Unlike the current findings, comfort activities were higher in beak trimmed pullets than intact beak ones^34^.

Feather pecking frequencies dramatically changed between different groups (*P* < 0.05). Feather pecking frequencies in trimmed ducks with scissors were lower than control and sprayed ones. Sprayed ducks with bitter almond oil recorded the highest FP frequencies. In consistent to our finding^26^observed that beak trimming with scissors notably decreased pecking frequency in mule ducks (*P* < 0.05) than control ducks. Feather pecking frequency was statistically lowered in trimmed Muscovy ducks with scissors than control ones^15^ and^10^ recorded that aggressive pecking behavior of control birds was significantly higher than trimmed ones Aggression significantly decreased in trimmed quails with hot blade method than birds those had intact beak^30^. In contrary to our results^20^ revealed that number of pulled and eaten feathers by hens that coated with 1% bitter almond oil was significantly lowered than uncoated feathers due to irritation and taste aversion of bitter almond oil thorough its odor^35^ concluded that downy feather spraying with bitter tasting substance as quinin solution in laying hen decreased the degree of feather pecking from severe to gentle feather pecking in adult stage. An early study indicated that coating feathers with distasteful substance such as quinin sulphate produced negative feedback on feather pecking and eating than sucrose solution that act as positive feedback in laying hens^36^. In chicken using almond oil enhanced the rate of avoidance learning to avoid food or water that had unfamiliar odor^13^. Increase the feather pecking frequency of bitter almond oil sprayed group may return to the attractive odor and taste of the oil that makes ducks approach each other and begin to explore the odor and the taste of the oil.

Stereotypic behaviors like litter scratching and object pecking showed a different response to beak trimming and feather spraying; litter scratching and object pecking bouts were increased in control ducks than in trimmed and sprayed ones. This may be attributed to beak trimming decreasing exploration activities. Our results were in an agreement with^26^ who revealed that pecking the environment in mule ducks was higher in control than in trimmed ducks. A previous study showed that trimmed Muscovy ducks with scissors significantly spent less time involved in exploratory pecking than control ones^4^^15^ observed wall pecking bouts increased in control birds than trimmed ducks, while there was not any significant change in floor pecking between the control group and trimmed ducks.

Feather condition was dramatically different between treatments. Trimmed ducks had better feather condition than control and sprayed ducks throughout the experiment period. The bitter almond oil sprayed group had a moderate feather score from 6 weeks old till the end of the study while the control ducks showed this score in the last 3 weeks of the trial. Beak trimming decreased the incidence of feather pecking resulted in good feather condition score. A lot of previous studies confirmed our results such as^26^ who observed that feather condition was improved in scissors trimmed mule ducks than control ones, moreover^30^ found that the plumage deterioration was notably reduced by beak trimming because aggression decreased, and the force of pecking was weakened by the beak trimming compared to intact beak birds. Trimmed laying hens with infrared or hot blade showed less aggressive pecking reduced severe FP and had better feather condition^33^. Contrary to the current study, cutting the bird’s beak didn’t make significant change in feather quality^37^, also spraying a bitter-tasting substance reduced severe feather pecking thus improved feather cover of laying hens^35^.

Cortisol level was significantly different between the 3 treatments; as control ducks had the lowest level of cortisol when compared to trimmed and sprayed ducks. Sprayed ducks were more stressed than other ducks, this may be attributed to the high frequency of feather pecking and feather deterioration and decreased feed intake^38^ reported that bill-trimmed pheasants had higher plasma corticosterone concentrations in comparison with control birds. On the other hand,^6,15^ recorded a non-significant difference in cortisol level between trimmed and control birds.

In the current study, the beaks of the control ducks had a normal average distribution of blood vessels, connective tissue and nerve cells, and no necrosis or degenerative changes in different beak tissues, unlike histopathological findings of beak of trimmed ducks with scissors that showed the disappearance of the epidermal layer, hyperplasia of the dermal tissue that leads to an increase in the amount of connective tissue (increase scar tissue formation and blood vessels) but nerve cells were few in number (no neuroma formation) compared with control group. The beak of bitter almond oil sprayed ducks revealed that connective tissue and blood vessels were prominent but of normal distribution and no changes in nerve cells compared with control one. Our results were coinciding with^39^ who found that infra-red bill trimming in laying hens not showed abnormal nerve regrowth up to 50-week post- trimming (no neuroma formation) so, it suggested that infra-red bill trimming did not result in chronic pain. Early study of^4^ on microscopic analysis of the bills of Muscovy ducks that trimmed with cold cutting cleared that the layer of connective tissue became much thicker and had an irregular orientation which is characteristic of scar tissue formation that leads to very little nerve regrowth into the bill stump (no evidence of neuroma formation) thus ducks not suffered from chronic pain also, bill stumps lacked blood vessels, while beak trimming of Pekin ducks with hot blade and hot searing showed that scar tissue that formed had many blood vessels as in our study^40^. Moreover, bill trimming of ducks resulted in no neuromas were found in the bill stumps^4,40^.

## Conclusion

In conclusion, beak trimming is a practice made by many poultry farms to control severe feather pecking and cannibalism. In our study, beak trimming of Muscovy ducks had no negative impacts on growth performance (body weight, body weight gain, feed intake and FCR). Bill trimming reduced the feather pecking bouts, resulting in good feather condition score and did not act as a stress factor. Feather spraying with bitter almond oil exerting a moderate adverse influence on duck performance, elevating pecking frequencies, which led to feather degradation and heightened stress among the birds. So, beak trimming with scissors at 3 weeks of age could be carried out without any adverse effect on performance and welfare of Muscovy ducks.

## Data Availability

The data presented in this study are available within the article.
